# The Eyes Have It: Sex and Sexual Orientation Differences in Pupil Dilation Patterns

**DOI:** 10.1371/journal.pone.0040256

**Published:** 2012-08-03

**Authors:** Gerulf Rieger, Ritch C. Savin-Williams

**Affiliations:** Department of Human Development, Cornell University, Ithaca, New York, United States of America; University of Sydney, Australia

## Abstract

Recent research suggests profound sex and sexual orientation differences in sexual response. These results, however, are based on measures of genital arousal, which have potential limitations such as volunteer bias and differential measures for the sexes. The present study introduces a measure less affected by these limitations. We assessed the pupil dilation of 325 men and women of various sexual orientations to male and female erotic stimuli. Results supported hypotheses. In general, self-reported sexual orientation corresponded with pupil dilation to men and women. Among men, substantial dilation to both sexes was most common in bisexual-identified men. In contrast, among women, substantial dilation to both sexes was most common in heterosexual-identified women. Possible reasons for these differences are discussed. Because the measure of pupil dilation is less invasive than previous measures of sexual response, it allows for studying diverse age and cultural populations, usually not included in sexuality research.

## Introduction

Recent research suggests that self-reported sexual orientation more strongly corresponds with sexual arousal to male or female sexual stimuli in men than in women [1]. In addition, the expression of bisexual arousal differs between the sexes. There is conflicting evidence regarding whether bisexual-identified men have a bisexual arousal pattern, but among women, a bisexual arousal pattern is most common among those who self-identify as heterosexual [2]–[4]. These findings, however, are based on measures of genital responses, which have potential limitations such as volunteer bias and different measurement devices for men and women. The present study introduces a measure that is less affected by these limitations: pupil dilation. We use this measure to examine previously suggested sex and sexual orientation differences in sexual response.

### Sex Differences in Sexual Response

Research has established that men and women react differentially to sexual stimuli, with men’s responses more influenced by the erotic information of stimuli and women’s responses more dependent on other, nonsexual aspects of the stimuli [5]–[7]. These findings concur with the proposal that in contrast to men, the sexual attraction patterns of women are less affected by a partner’s sex and more by cultural, social, and situational variables [8]–[10]. These variables include pair bonds, attachment history, educational experiences, religious attitudes and beliefs, and acculturation [5], [6]. Through socialization, these experiences alter women’s capacity for sexual response, leading to greater variability in sexual arousal [11]. The consequence, according to Peplau [6], is that both sexual and nonsexual experiences are crucial for shaping women’s sexual orientation and attraction, including the potential for some women to change their sexual attraction to men and women over time and across contexts.

Baumeister [8] proposed that the underlying mechanism for this sex difference in the variability of sexual attraction has an evolutionary basis. Baumeister writes on page 347 that sexuality evolved to “suit the reproductive contingencies of males and females so as to maximize the passing on of each person’s genes.” Men evolved to be strongly driven by innate motivational patterns that are relatively constant and unchanging across time and situations. Women evolved to be responsive to the male sex drive and to be flexible in whether and what situations they respond to the male drive. From this perspective, female sexuality, more than male sexuality, adapts to changing circumstances. This difference influences women’s sexuality to be more responsive to environmental circumstances.

As a reflection of this general sex difference, the relationship of genital arousal to either male or female sexual stimuli with self-reported sexual orientation is considerably stronger in men than in women [2], [12]. Most men are exclusively aroused to the sex consistent with their reported sexual orientation; for example, most heterosexual men are almost exclusively aroused to women and most homosexual men are almost exclusively aroused to men. In contrast, women’s sexual orientation is poorly reflected in their genital response because they respond with substantial arousal to both sexes [2], [12]. These results suggest a substantial difference between the sexes in the organization of sexual orientation.

If sex differences in sexual orientation and arousal are robust and not restricted to measures of genital response, then other measures of sexual arousal should also indicate them. The present research used pupillary response as a measure of sexual arousal. Thus, our first hypothesis was that the correspondence of pupil dilation to male or female sexual stimuli with self-reported sexual orientation would be stronger in men than women.

### Sexual Orientation Differences in Sexual Response

In addition to the aforementioned sex differences, genital arousal measures illuminate differences in sexual response, depending on a person’s sexual orientation. Although most men show nearly exclusive sexual arousal to one sex, a substantial minority of men identifies as bisexual and therefore might be expected to display strong arousal to both sexes. Some research suggested, however, that bisexual men exhibit almost exclusive sexual arousal to either men or women, but not to both [3], [13]. According to this research, bisexual men are similar to most other men in that they show substantial arousal to only one sex. More recent research by Rosenthal et al. [4], however, indicated that more stringent recruitment methods produce self-reported bisexual men who show a bisexual genital arousal pattern. For example, bisexual-identified participants were recruited through websites that cater towards men who seek sexual relations with both men and women. According to another study, bisexual men have a genital arousal pattern that can be described as bisexual [14], although other interpetation of these data was given [15]. In total, there is some but inconclusive support for the hypothesis that bisexual men’s sexual response is a reflection of their self-reported sexual orientation.

The present study used recruitment methods similar to those employed by Rosenthal et al. [4], but used pupillary response instead of genital response to measure sexual arousal. Thus, our second hypothesis was that bisexual men would show greater pupil dilation to both male and female sexual stimuli compared to heterosexual and homosexual men.

We previously noted that men and women differ in their sexual attraction and behavioral patterns because environmental and innate factors influence the sexes differently [8]–[10]. Other authors have pointed out that innate factors account for sexual orientation differences in sexual attraction [16], [17], although some effective socialization processes can be theorized. For example, cross-cultural research compared the same-sex sexual behaviors of heterosexual men in Turkey, Thailand, and Brazil [18], [19]. The incidence of same-sex sexual behavior was greater in the first two groups, but especially among working class Turkish heterosexual men. According to Cardoso, because most Turkish women of this social class are sexually unavailable, men are more likely to engage in same-sex behavior. Hence, the finding that some men show sexual arousal to both sexes, or engage in sexual behavior with both sexes, may be due to cultural and social class influences.

Unlike most men, many women show substantial sexual arousal to both sexes. This general pattern, however, is moderated by women’s sexual orientation and is most common among heterosexual women, who show similarly strong sexual arousal to male and female sexual stimuli. In contrast, this pattern is less common among homosexual women, who show more sexual arousal to female stimuli and somewhat less sexual arousal to male stimuli [2], [12]. In this sense, homosexual women show more male-typical sexual arousal patterns compared to other women. This observation led to the third hypothesis of this study: Homosexual women would show greater pupil dilation to female stimuli, and less to male stimuli, and heterosexual women would show more equal dilation to both sexes.

### Pupil Dilation as a Measure of Sexual Response

Potential limitations in assessing sexual orientation by genital arousal have been pointed out [20]. First, a substantial number of people are reluctant to participate in a study that assesses genital response [12], [21] and those who do might represent an unusual population in unknown respects, thus creating results that may not apply broadly. Second, genital arousal is measured differently in the two sexes. In men, a common instrument measures penile circumference; in women, a common instrument assesses vaginal pulse amplitude [22]. Thus, it cannot be concluded with confidence whether distinctions between men and women are due to actual sex differences in sexual arousal or to dissimilarities of measurement. Third, some people can willingly suppress genital arousal to sexual stimuli, which affects the accuracy of the measure [23], [24].

One measure that is less affected by these limitations is pupil dilation. Participants are arguably less likely to opt out of an experiment that assesses their eye gaze rather than their genital response. Across both sexes, response of the same organ (the eye) can be measured with identical instruments. Furthermore, pupil dilation to stimuli indicates activation of the autonomic nervous system [25], [26]. This system is associated with many automatic processes such as perspiration, digestion, blood pressure, and heart rate [27]. For this reason, pupil dilation has been used as indicator of automatic response, for example, in studies of implicit reaction and cognitive load [28], [29]. Other research suggests that pupil dilation can reflect automatic attention, or attention that is likely not in the conscious control of participants [30]. It is therefore unlikely that participants suppress pupil dilation to stimuli they are sexually attracted to. Pupil dilation patterns could therefore reflect, with high sensitivity, automatic attention related to sexual attraction and sexual orientation.

One study previously employed pupil dilation to measure sexual orientation [31]. This study investigated the dilation patterns of five heterosexual men and five homosexual men to images of nude men and women. Pupil dilation patterns corresponded highly with sexual orientation. However, the majority of participants was associated with the investigators, which could have affected results. To date, there has been no known attempt to confirm these findings with a larger and more representative sample.

Other than for basic research, pupillary responses as a measure of sexual orientation have been employed by the Canadian Government between the 1950s and 1970s. The goal of this program was to detect homosexual individuals, who were at the time considered a national risk. These experiments were noted to be fallacious [32], [33] because of several methodological limitations [34]. The authorities had great difficulty recruiting both heterosexual and homosexual volunteers, which likely meant that they did not have sufficient statistical power for comparisons. Only heterosexual and homosexual identities were taken into account; thus, if bisexual individuals participated, they were not noted as such, which could have distorted results. The experimenters had problems adjusting for variation in pupil size. Without this adjustment, individual responses, including those of individuals with different sexual orientations, cannot be accurately compared. It is unknown how consistent the distance was between the pupil and the camera that captured its image. Without this information it is problematic to compare responses across individuals. Many pupillary changes that occurred were in a range that was less than one millimeter. Because these changes were measured by hand, it is likely that a large amount of error was added to the data. Sexual stimuli varied in degree of luminance, which could have caused pupillary responses unrelated to the individual’s sexual attraction to stimuli. These experiments never efficiently produced results, despite possessing some unique technological qualities.

The present research is free of the vast majority of the aforementioned limitations. Over 320 participants agreed to take part in this study and they had multiple options to indicate their sexual identity, ranging from exclusively heterosexual, to bisexual, to exclusively homosexual. An infrared gaze tracker automatically recorded for each participant both pupil size and degree of dilation. Data were standardized within participants to allow accurate comparisons across participants. The distance of participants to the camera was kept constant. The measurement of pupil size was in terms of camera pixel occluded by the pupil; thus, the measure of pupillary response did not rely on an assessment by hand. In addition, the present research had some, albeit crude, control over the luminance of stimuli. Overall, compared to the studies sponsored by the Canadian Government [33], [34], the present research employed methodologies that were considerably more advanced and precise.

### Correspondence of Measures

Because pupil dilation has rarely been used in systematic research as an indicator of sexual orientation, it is important to assess its validity. Other research has measured time spent viewing male or female stimuli to examine sex and sexual orientation differences in sexual response [35]–[39]. There may be correspondences among pupil dilation to stimuli, viewing time of stimuli, self-reported sexual attraction to stimuli, and self-reported orientation. These correspondences would point to the general validity of all measures. Thus, the fourth hypothesis was that pupil dilation would be positively related to other measures of sexual attraction, and all measures would be positively related to self-reported sexual orientation.

### Summary of Hypotheses

Based on previous research using other measures of sexual response, the following hypotheses were tested:

1. The correspondence of pupil dilation to male or female sexual stimuli with self-reported sexual orientation will be stronger in men than women.
2. Bisexual men will show greater pupil dilation to both male and female sexual stimuli compared to heterosexual and homosexual men.
3. Homosexual women will show greater pupil dilation to female stimuli and less to male stimuli, and heterosexual women will show more equal dilation to both sexes.
4. Pupil dilation to sexual stimuli will correspond with time spent viewing these stimuli, self-reported sexual attraction to these stimuli, and self-reported sexual orientation.

## Methods

### Participants

Advertisements were placed on several websites for members of a Northeast university. We also recruited from a web forum where men sought both men and women for sexual reasons. The latter method was previously successful in recruiting bisexual-identified men [4], a group less prevalent than other men [40]. A total of 165 men and 160 women indicated their sexual orientation identity on a 7-point scale, ranging from “exclusively straight” to “bisexual” to “exclusively gay/lesbian.” Table 1 shows the number of participants for each sexual orientation identity by age and ethnicity.

The average age (SD) was 23.36 (6.62) years in men and 27.70 (6.78) years in women. The most common ethnicity was Caucasian, 64% and 69%, respectively. The second most common group (10%) indicated mixed ethnicities, and the remainder identified as Asian, African-American, Hispanic, and Native-American. Across sexual orientation identities there were differences in age and proportions of being Caucasian (Table 1). These differences did not significantly affect patterns of results reported below.

**Table 1:** Distribution of Sexual Orientation Identities, Ages, and Ethnicities.

	Men (N = 165)						
Variable	Sexual Orientation Identity						
	Exclusively Straight	Mostly Straight	Bisexual Leaning Straight	Bisexual	Bisexual Leaning Gay	Mostly Gay	Exclusively Gay
Number	31	24	15	10	21	33	31
Average Age (SD)	20.83 (2.79)	23.58 (7.00)	25.67 (7.51)	28.67 (12.09)	25.50 (6.96)	22.76 (5.65)	24.00 (7.16)
Percentage Caucasian	65	63	67	67	59	64	77
	**Women (N = 160)**						
**Variable**	**Sexual Orientation Identity**						
	**Exclusively Straight**	**Mostly Straight**	**Bisexual Leaning Straight**	**Bisexual**	**Bisexual Leaning Lesbian**	**Mostly Lesbian**	**Exclusively Lesbian**
Number	34	27	11	17	16	32	23
Average Age (SD)	21.88 (6.73)	21.67 (4.29)	22.36 (4.25)	24.41 (6.92)	20.81 (2.07)	26.31 (9.46)	24.87 (5.17)
Percentage Caucasian^1^	47	63	55	76	69	84	91

*Note*. ^1^Caucasian was the most common ethnicity in men (65%) and women (69%). The second most common group indicated mixed ethnicities in men (11%) and women (9%), and the remainder identified as Asian, African-American, Hispanic, and Native-American.

### Measures

#### Self-reported sexual orientation

Participants reported their sexual orientation identities, attractions, fantasies, and infatuations on four Kinsey-type scales [41]. These measures were highly correlated in men (all *p’s* <.0001, all *r’s* ≥.95) and women (all *p’s* <.0001, all *r’s* ≥.92), and were averaged within participants. For this composite, a score of 0 indicated an exclusively heterosexual orientation and a score of 6 an exclusively homosexual orientation.

#### Stimuli

Each stimulus was a 30-second video showing either a naked male or female model masturbating. In total, 12 male stimuli and 12 female stimuli were selected from a large pool of videos drawn from sites on the Internet. In a pilot study, heterosexual and homosexual men and women rated these videos on the models’ sexual appeal, and the most appealing stimuli were used in the present study. Two 1-minute videos, showing landscapes, were taken from a nature documentary for creation of neutral stimuli. Luminance of stimuli was set to equal thresholds using FinalCut Pro.

#### Pupil data

An SR Research Remote infrared gaze tracker recorded participants’ eyes. The program EyeLink computed pupil area as the number of the tracker’s camera pixels occluded by the pupil. *Pupil dilation and constriction* was based on changes of pupil area when viewing sexual stimuli as compared to neutral stimuli. Given that results are presented with respect to dilation, we refer to this measure as *pupil dilation*.

*Viewing time* was measured as percentage of time participants looked at a male or female sexual stimulus.

### Procedure

Cornell University’s Institutional Review Board for Human Participants (at the Office of Research Integrity and Assurance) specifically approved this study. Participants provided written informed consent once they arrived at the university lab. They were seated in a dimly lit room facing a monitor with a screen resolution of 1024 by 768 pixels. The gaze tracker was placed underneath the screen and collected data every two milliseconds with a 16 mm lens focused on participants’ preferred eye. Participants’ heads rested on a mount 500 mm from the lens, and the head’s exact position was automatically recorded by measuring the distance from the lens to the forehead.

Stimuli were presented in two modules. The first module was used for collection of pupil dilation data. Participants watched a neutral stimulus, followed, in random order, by the sexual stimuli. After each stimulus, participants answered three questions regarding, in random order, how sexually attractive they found the person, how sexually appealing they found the person, and how much they would like to date this person. Participants answered each question with a 7-point scale ranging from “not at all” to “average,” to “very much.” These questions were written on the screen, set against a white background. Participants used a mouse to answer questions, by clicking at the number of their choice. This visual display was the same before each subsequent stimulus. Participants were instructed to give answers without thinking too hard, but they had as much time as needed to answer questions. The vast majority of participants answered these questions within three seconds. After they answered the last question, the next stimulus was presented.

Immediately following the first module, the second module began with a neutral stimulus. Then, two stimuli were presented simultaneously. Half of these paired stimuli showed the male to the right of the female; the other half had the opposite presentation. Paired stimuli were shown in random order. This module was chosen for the collection of data regarding time spent viewing men or women. After each stimulus pair, participants responded to three questions, in random order, about which person they found more sexually attractive, more sexually appealing, and would more like to date. Participants answered each question with a 7-point scale ranging from “very much the left,” to “equal,” to “very much the right”.

Finally, participants completed a questionnaire with demographic information and sexual orientation and received payment. The total procedure took, on average, 45 minutes.

### Data Reduction

Because each stimulus was presented immediately after the questions for the previous stimulus, there was a chance that undesired factors influenced pupillary response to stimuli. Specifically, degree of attention to the previous stimulus and its questions could result in cognitive load and, thus, affect pupillary response to the subsequent stimulus. To avoid such influences, data analyses were restricted to the last 10 seconds of each stimulus. The subsequent results were virtually identical, regardless of whether the full stimuli length or the last 10 seconds were used for analyses. However, restricting analyses to the last 10 seconds yielded, in general, marginally stronger effect sizes.

For each participant, pupil size data were averaged in two steps, within stimulus and across stimuli of the same type. Specifically, for each participant, pupil size data were first averaged across the last 10 seconds of each stimulus. Averaged pupil size was multiplied with averaged head distance for each stimulus, to account for heads moving somewhat between stimuli. There is no consensus as to the most appropriate technique of measuring and analyzing pupil size data [42]. We computed within each participant z-scores of pupillary response because pupils vary in size and in degree of dilation. For men and women, pupil size data were highly reliable for all male sexual stimuli, female sexual stimuli, and neutral stimuli (all *Cronbach’s α’s* ≥.96, respectively). This means, for example, if participants showed increased pupillary response to a male sexual stimulus (as compared to both a neutral stimulus and other participants), they were likely to respond in this way to all other male sexual stimuli. Because of these consistent responses within stimuli types, we computed, for each participant, three mean values reflecting average pupil dilation to male sexual stimuli, female sexual stimuli, and neutral stimuli. Whenever pupillary response to sexual stimuli was used in analyses, we first subtracted response to neutral stimuli. Thus, positive scores of these variables indicated increased pupil dilation to sexual stimuli as compared to neutral stimuli; a negative score would indicate constriction compared to neutral.

Viewing time was computed such that higher numbers indicated higher percentage of time viewing the same sex. These percentages were highly reliable across paired stimuli (*Cronbach’s α* ≥.96 in both sexes) and averaged within participants.

The three ratings of stimuli were reliable within each stimulus, across all male stimuli, across all female stimuli, and across all male-female stimuli (all *Cronbach’s α’s* ≥.95). Thus, for each participant and each stimulus type, an average was computed across ratings. These averages represented, respectively, participants’ general self-reported sexual attraction to stimuli of the same sex, sexual attraction to stimuli of the other sex, and, from the second module, a contrast reflecting sexual attraction to the same or other sex.

## Results

### Sex Differences in Sexual Orientation and Pupil Dilation

Our first hypothesis stated that the relation of pupil dilation to male and female stimuli with self-reported sexual orientation would be stronger in men than women. For both men and women, there was a strong and inverse correlation of pupil dilation to same-sex stimuli with pupil dilation to other sex stimuli, *p*<.0001, *r = *−.77 and *p*<.0001, *r = *−.77, respectively. We therefore created a contrast by subtracting participants’ dilation to the other sex from dilation to the same sex. For this pupil dilation contrast a positive score represented more dilation to the same sex and less dilation to the other sex, a negative score represented more dilation to the other sex and less dilation to the same sex, and a score of zero represents equal dilation to both sexes. For each sex, this contrast was regressed against self-reported sexual orientation by conducting a regression analysis that included both the linear and curvilinear effect of sexual orientation. The curvilinear effect was included because, for example, the relationship of sexual orientation with pupil dilation to the preferred sex may not be equally strong in heterosexual and homosexual participants.

**Figure 1:**
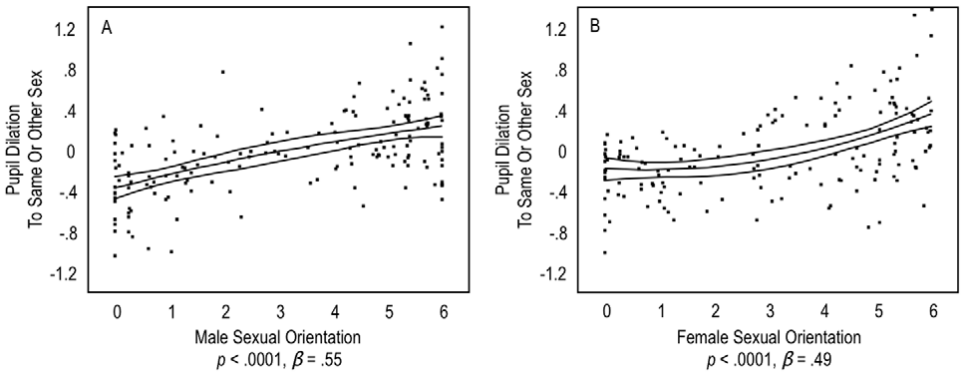
Pupil dilation to the same sex and the other sex. Panel A shows men’s responses and Panel B shows women’s’ responses. Y Axes reflect z-scores within participants: positive numbers indicate dilation to the same sex, and negative numbers indicate dilation to the other sex. X Axes reflect self-reported sexual orientation: 0 represents an exclusive heterosexual orientation, 3 an even bisexual orientation, and 6 an exclusive homosexual orientation. Triple lines represent regression coefficients with 95% confidence intervals. Dots represent participants’ average scores. *β’s* are standardized coefficients for linear effects.

In men, the linear relationship of the pupil dilation contrast with sexual orientation was significant, *p*<.0001, *β* = .55. (The effect size *β* is the standardized regression coefficient and can be interpreted similarly to a correlation coefficient.) Heterosexual men dilated most to the other sex, homosexual men dilated most to the same sex, and bisexual men dilated more equally than other men to both sexes (Figure 1A). For heterosexual, bisexual, and homosexual women, pupil dilation to the same or other sex was similarly related to their sexual orientation (Figure 1B), *p*<.0001, *β* = .49.

The curvilinear effects of sexual orientation on pupil dilation was not significant in men, *p* = .49, *β* = -.05. Figure 1A shows that exclusive heterosexual men (Kinsey Score of 0) and homosexual men (Kinsey Score of 6) dilated almost equally strongly to their preferred sex, yielding the aforementioned linear effect. In contrast, in women, there was indication of a curvilinear effect. Figure 1B shows that exclusive heterosexual women had significantly greater pupil dilation to the other sex than to the same sex; however, in magnitude, exclusive homosexual women dilated more to the same sex than the other sex. This difference yielded, within women, a significant curvilinear effect, *p* = .03, *β* = .15. The finding that homosexual women had more orientation-specific dilation patterns than other women is in line with the third hypothesis investigated below.

A subsequent multiple regression analysis tested for sex differences in these effects. Pupil dilation the same sex or other sex was predicted by the linear effect of sexual orientation, the curvilinear effect of sexual orientation, and by participant’s sex (converted into a numerical variable with values 0 for males and 1 for females). Other predictor variables included the interactions of the linear and curvilinear effects of sexual orientation with sex. These interactions tested whether the sexes differed in these effects. Results of this regression analysis indicated no significant sex difference for the linear relation of pupil dilation with sexual orientation, *p* = .82, *β* = .01. There was, however, a significant sex difference for the curvilinear relationship, *p* = .04, *β* = .18. This result suggested that the curvilinear relationship is more common in women than men; this sex difference is reflected in the comparisons of Figures 1A and 1B.

Figure 1 also shows that within exclusive heterosexual men and women (Kinsey Score of 0) there was an additional sex difference. Exclusive heterosexual men showed greater pupil dilation to the other sex than same sex compared to exclusive heterosexual women. We computed an independent-sample *t*-test, comparing exclusive heterosexual men and women on their pupil dilation to the other sex over the same sex. This sex difference was marginally significant, *p* = .08, *d* = 0.66.

### Pupil Dilation Patterns within Men

Our second hypothesis stated that bisexual men would show a bisexual dilation pattern. We computed two new variables, one for participants’ pupil dilation toward the more arousing (dilation-triggering) sex and one for the less arousing sex [3]. We assumed the level of pupil dilation toward the more arousing sex to be similar across men of different sexual orientations. The crucial analysis concerned the less arousing sex; if bisexual men have bisexual pupil dilation patterns, then they should show significantly greater pupil dilation toward the less arousing sex (whichever that sex happens to be) than do either heterosexual or homosexual men.

**Figure 2:**
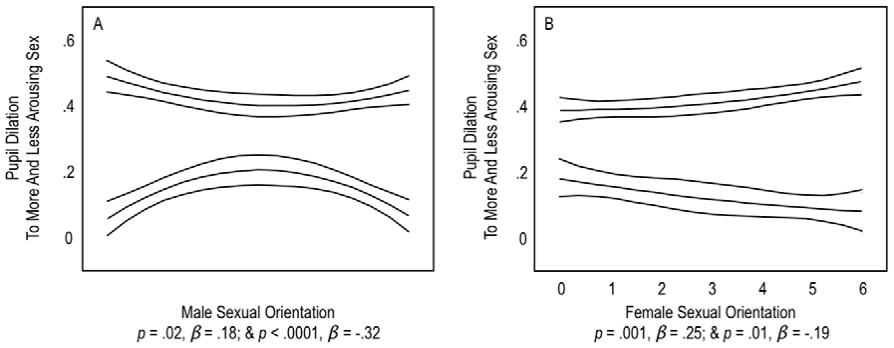
Pupil dilation to the more arousing sex and less arousing sex. Panel A shows men’s responses and Panel B shows women’s’ responses to the more arousing sex (i.e., more dilation-eliciting sex; upper lines) and the less arousing sex (lower lines). Y Axes reflect, within participants, z-scores compared to a neutral stimulus. X Axes reflect self-reported sexual orientation: 0 represents an exclusive heterosexual orientation, 3 an even bisexual orientation, and 6 an exclusive homosexual orientation. Triple lines represent regression coefficients with 95% confidence intervals. *β’s* are, for men, standardized coefficients for curvilinear effects, and, for women, standardized coefficients for linear effects.

We conducted a multiple linear regression analysis predicting men’s pupil dilation to the less arousing sex by the linear and curvilinear effect of sexual orientation. Results confirmed this hypothesis. Figure 2A indicates that, compared to a neutral stimulus, men with bisexual orientations (Kinsey Scores of 2, 3, or 4) displayed significantly greater pupil dilation to the less arousing sex than either heterosexual or homosexual men. This curvilinear effect was significant, *p*<.0001, *β* = −.32. Thus, with respect to pupil dilation, bisexual men had bisexual responses. The linear effect of men’s sexual orientation on their pupil dilation to the less arousing sex was not significant, *p* = .26, *β* = .09.

To obtain a substantial sample of bisexual men, we recruited from a web forum where men sought sex with men and women. Thus, our bisexual men may have had bisexual pupil dilations because they presented as highly sexual men, in general, and not because they had responses specific for their identity. We re-analyzed data after excluding the 24 bisexual men recruited from the forum and kept the 22 bisexual men recruited similarly to other participants. Results remained similar; for example, bisexual men showed bisexual pupil dilation patterns before and after exclusion, *p*<.0001, *β* = −.32, and *p* = .02, *β* = −.25, respectively. Differences between bisexual men and other men were therefore not strongly based on recruitment venue.

Notably, Figure 2A also shows that bisexual men dilated significantly *less* to their more arousing sex, compared to both heterosexual and homosexual men, *p* = .02, *β* = .18. This effect was weaker, in magnitude, than bisexual men’s greater pupil dilation to their less arousing sex, compared to other men, *p*<.0001, *β* = −.32.

### Pupil Dilation Patterns within Women

Our third hypothesis indicated that homosexual women would have greater pupil dilation to their more arousing sex and less dilation to the less arousing sex compared to other women. In contrast, heterosexual women would show more equal pupil dilation to both sexes.

We conducted two multiple linear regression analyses predicting either women’s pupil dilation to the more arousing sex or their pupil dilation to the less arousing sex by sexual orientation. Figure 2B shows that pupil dilation to the more arousing sex, compared to a neutral stimulus, was stronger in homosexual women than in other women, *p* = .001, *β* = .25. By contrast, dilation to the less arousing sex was weaker in homosexual women than in other women, *p* = .01, *β* = −.19. Thus, among women, homosexual women showed the strongest contrast in pupil dilation to the more versus less arousing sex.

Figure 2B also shows that for bisexual women (Kinsey Scores of 2, 3, or 4) the contrast in pupil dilation to the more versus less arousing sex was stronger than the contrast found in heterosexual women, but less strong than the contrast found in homosexual women. When compared to the heterosexual women only, their pupil dilation contrast was significantly stronger, *p* = .003, *β* = .24; when compared to the homosexual women only, their pupil dilation contrast was significantly weaker *p* = .009, *β* = −.23, respectively. Thus, in their pupil dilation to the more and less arousing sex, bisexual women were in an intermediate position between heterosexual and homosexual women.

Finally, Figure 2 shows that for exclusive heterosexual men and women, pupil dilations to the more and less arousing sex were different. Heterosexual men dilated strongly to the more arousing sex and little to the less arousing sex, compared to neutral stimuli. By contrast, heterosexual women dilated less to the more arousing sex, compared to both neutral stimuli and men, and they dilated more to the less arousing sex, compared to both neutral stimuli and men. Hence, heterosexual women dilated more strongly to the less arousing sex than did heterosexual men, *p* = .01, *d* = 1.00.

### Consistency across Measures

Our fourth hypothesis regarded the correspondence of measures. Table 2 shows the correlations across pupil dilation, viewing time of same-sex or other-sex stimuli, self-reported sexual attraction toward stimuli, and self-reported sexual orientation. In general, these correlations were modest and significant, suggesting that all measures, including pupil dilation, are indicators of sexual attraction and orientation.

**Table 2:** Correlations between Pupil Dilation, Viewing Time, Self-Reported Attraction to Sexual Stimuli, and Self-Reported Sexual Orientation across Male (N = 165, above Diagonal) and Female (N = 160, below Diagonal) Participants.

Measure	Pupil Dilation To Same Sex	Pupil Dilation To Other Sex	Viewing Time^1^	Attraction to Same Sex Stimuli	Attraction to Other Sex Stimuli	Sexual Attraction Contrast^1^	Sexual Orientation^1^
Pupil Dilation To Same Sex		−.77***	.51***	.47***	−.39***	.53***	.50***
Pupil Dilation To Other Sex	−.77***		−.54***	−.44***	.46***	−.57***	−.57***
Viewing Time^1^	.45***	−.49***		.77***	−.74***	.93***	.91***
Sexual Attraction to Same Sex Stimuli	.35**	−.39***	.69***		−.35***	.80***	.75***
Sexual Attraction to Other Sex Stimuli	−.36***	.33**	−.58***	−.14†		−.77***	.76***
Sexual Attraction Contrast^1^	.39***	−.43***	.89***	.76***	−.61***		.95***
Sexual Orientation^1^	.44***	−.45***	.82***	.67***	−.60***	.90***	

*Note.* ^1^Higher scores indicate stronger response or attraction to the same sex and less to the other sex.

† *p*<.10.

** *p*<.001.

*** *p*<.0001.

One of the strongest correlations was between self-reported sexual orientation and time spent viewing same-sex or other-sex stimuli (Table 2). The weakest correlations were found between self-reported sexual attraction to same-sex stimuli and sexual attraction to other-sex stimuli.

The average absolute correlation was.64 in men and.55 in women. A repeated measures *t*-test, with calculated sex differences in correlations within pairs of variables, suggested that the magnitude of absolute correlations was significantly stronger in men than women, *t*(20) = 7.83, *p*<.0001, *d* = 1.43.

## Discussion

Results suggested that pupil dilation is a significant indicator of sexual orientation. Within heterosexual men and women, findings confirmed hypothesized sex differences in sexual response. Furthermore, results indicated that bisexual men have bisexual dilation patterns, and homosexual women have male-typical dilation patterns.

### Sex Differences

The overall relationships of sexual orientation with pupil dilation and other measures were stronger in men than in women (Table 2). Other research has reported corresponding sex differences [7], [43]. The simple relationship of sexual orientation with pupil dilation patterns to sexual stimuli was not significantly stronger in men than women, largely because among homosexual women the relationship of their sexual orientation with dilation patterns was similar to the relationship seen in men.

Among heterosexual participants, substantial sex differences in pupil response were found (Figures 1 & 2). Compared to heterosexual men, heterosexual women showed a more equal sexual response to male and female stimuli. This sex difference is consistent with previously reviewed theoretical writings [5], [6], [8], [9] and documented sex differences in genital response [1]. Baumeister [8] argued that the sexes evolved to differ in their sexual responsiveness, and this is an adaptation to the sexual behavior of the other sex. One hypothesis related to Baumeister’s proposal is that sexual response has different biological functions for men and women [2]. For men, an important function is to facilitate erection and penetration; for women, to facilitate lubrication and prevent genital injury in case of penetration. Support for this hypothesis is derived from both cross-species and cross-cultural comparisons. Forced copulation in several species [44]–[46] and in most human societies [47], [48] indicate that it may have occurred throughout human evolution [49]. Because forced copulation can lead to genital trauma [50], the female response to any sexual stimulus may have evolved in part to mitigate this risk. Related reasons have been discussed regarding how and why mate choice [51] and sexual arousal [1] differ between the sexes.

When proposing these evolutionary hypotheses for sex differences in sexual arousal, we focus on heterosexual men and women. The vast majority of people is heterosexual [40], [52], and a sexual orientation towards the other sex is likely promoted by evolutionary mechanisms; thus, a focus on heterosexual individuals is justified. From an evolutionary perspective, exclusive homosexuality as found in humans is a conundrum [53]. Some research has suggested that, at least in men, the decreased fecundity of homosexual males is counter-balanced by the increased fecundity of their relatives [54]. Why such a balancing mechanism might exist and how it would relate to general sex differences in attraction and arousal is still unknown.

### Sexual Orientation Differences In Men

Bisexual men displayed bisexual pupil dilation patterns consistent with the finding that bisexual men show bisexual genital arousal [4]. The previous conclusion that bisexual men do not show such arousal [3] may have been based on a sample that identified as bisexual for reasons other than having strong sexual responses to both sexes. We also point to other findings that only a few men report attraction to both sexes [40], [52]. Men who have *both* bisexual identities and bisexual responses may therefore constitute an uncommon group that differs in some aspects from bisexually-identified men who have sexual responses to only one sex. It is possible, for example, that some men identify as bisexual not because they show bisexual arousal but because they have distinct personalities that open them to a variety of sexual experiences, including sexual experiences with the less preferred sex [55], [56].

Notably, bisexual men in the present study dilated somewhat *less* to their more arousing sex, compared to both heterosexual and homosexual men (Figure 2A). Blanchard, a leading figure in psychophysiological research on sexuality [57], [58], noted in unpublished data a similar pattern with respect to genital arousal. Perhaps, some bisexual men need further input other than visual stimulation to achieve maximum sexual arousal to their preferred sex. Possibilities include a need for higher levels of tactile stimulation or proprioceptive feedback from their own sexual behavior in order to show maximum sexual response (Blanchard, personal communication).

### Sexual Orientation Differences In Women

The present study confirmed the hypothesis that homosexual women have more male-typical sexual responses when compared to other women [2]. Homosexual women are, on average, more masculine than other women not only in motor behavior, voice pattern, facial features, and appearance (both self-reported and perceived by others) but also in their self-concepts and interests [59]–[62]. Prospective studies suggest that differences in masculinity-femininity appear in early childhood and prior to the development of an adult sexual orientation identity [63], [64]. Cross-culturally, sexual orientation differences in masculinity-femininity are not restricted to Western cultures but are found in other societies [65], [66]. These observations lead to the hypothesis that non-social and non-cultural factors are important for the co-development of sexual orientation with masculinity-femininity.

Social factors are certainly important for the development of some gender-typed behavior [67], but there is little if any evidence that they affect the co-development of sexual orientation with masculinity-femininity [17]. To date, prominent candidates for this co-development include prenatal gonadal influences [68], [69] and genetic influences [70], [71]. If these factors account for a general association of homosexuality with masculinity in women, they may also explain the present finding that, compared to other women, homosexual women had male-typical sexual responses.

### Utility of Pupillary Response

Pupil dilation was, in general, a robust indicator of sexual orientation. Pupillary response, viewing time, and self-reported sexual attraction to stimuli correlated with each other and with sexual orientation (Table 2). We note that these measures have their limitations. Pupillary response can be influenced by factors in addition to sexual orientation, including luminance and cognitive load [28], [29]. Aspects of viewing time can be under the conscious control of participants [72], [73], and the same is the case for self-reported attraction. Yet, despite their different methodological limitations, the present measures corresponded with each other, which supports the general validity of all measures and points to a core factor of sexual attraction and orientation.

In the present study the simple relationship of male self-reported sexual orientation with pupil dilation to the same or other sex (*r* = .57) was weaker in effect than corresponding effects of other research based on genital arousal measures, with *r’s* ranging from.77 to.83 [2], [3]. In this sense, the assessment of genital arousal measures appears to be the more precise measure of the two. Yet, because the assessment of genital response is more invasive than the assessment of pupillary response, the latter is more appealing for a wide range of participants. Moreover, in combination with stimuli that are not sexually explicit (e.g., images of dressed men and women), pupil dilation could be used with populations for which it would be problematic to use genital arousal measures, such as the study of sexual orientation among minors or in traditional cultures.

In the present study, the relationship of female self-reported sexual orientation with pupil dilation to the same sex or other sex (*r* = .47) was larger in effect than corresponding effects in other studies based on genital arousal, with *r’s* ranging from.21 to.24 [2], [12]. Perhaps, pupil dilation is a more sensitive indicator of female sexual orientation than vaginal pulse amplitude. Future research should compare these measures systematically in order to investigate which is the more robust correlate of female sexual orientation.

Viewing time of male or female stimuli was more strongly related to self-reported sexual orientation than was pupil dilation (Table 2). As aforementioned, because aspects of viewing stimuli are controllable by participants [72], [73], similar to self-reports, their strong correspondence would be expected. In contrast, pupil dilation is likely a measure of autonomic or unconscious response [25], [30]. Thus, pupil dilation may be the more desirable measure in future research, despite its weaker correlations with self-reported sexual orientation.

We emphasize that in the present study, sexual orientation differences in pupillary response were significant, *on average*, and with effects that were moderate to strong in magnitude, but not perfect, using the guidelines suggested by Cohen [74]. Consequently, not *every* participant’s sexual orientation was correctly classified, based on his or her pupil dilation to the same or other sex. Figure 1 illustrates that an observable amount of variability in pupil dilation was unrelated to the participant’s sexual orientation.

### Limitations

The present study had several methodological limitations. Pupils dilate to factors other than to how arousing stimuli are, such as the amount of cognitive load they produce and the stimuli’s level of luminance and contrast [28], [29]. In the present study, average luminance of stimuli and contrast was not set equal across stimuli. This was attempted but the resulting stimuli appeared extremely distorted and could not be used, which is not an uncommon problem when adjusting these factors, especially in videos.

We therefore selected videos that had, subjectively, similar luminance and then applied the filters on luminance thresholds. Although this procedure has clear limitations, it is unlikely that they resulted in confounded findings. Figure 1A shows that heterosexual and homosexual men dilated almost equally strong to their preferred sex, and bisexual men showed almost equal dilation to both sexes. Such patterns were consistent with our expectations about differences in sexual orientations. These patterns were, in general, similar in women, although we point to hypothesized sex differences in this effect. Given these systematic differences, results are less likely due to a lack of control of luminance or other factors. In fact, given the lack of control, it is likely that there was a certain level of noise in our data and that actual sexual orientation differences in pupillary response would have been stronger in effect if we had full control over all visual factors.

Another limitation was that neutral stimuli were presented twice, one time each before a block of stimuli was presented. A refined methodology would include a neutral stimulus that is presented before each sexual stimulus and would be used as specific comparison for this stimulus.

### Future Research

The present study suggests that measures of pupil dilation and genital arousal show similar patterns related to sex and sexual orientation. This proposal is indirect, however, because only pupil dilation was assessed in the present study. An important future study is to compare participants whose pupillary response and genital response are simultaneously assessed with participants who were recruited for a study on only pupil dilation. A comparison of these groups could systematically assess whether type of measure leads to ascertainment biases and affects patterns of results.

Personal and interpersonal factors other than sexual attraction may affect pupil dilation to stimuli of the same sex or other sex. For example, participants may dilate in response to comparing themselves, on a physical or social level, more with stimuli of their own sex than with the other sex (e.g., by asking whether they are better looking than the stimulus). Similarly, perceived personality characteristics of men and women used for stimuli could affect pupillary responses. Future research should assess how such effects compare, in magnitude, to the effects of sexual orientation on pupillary response.

### Conclusions

Findings from the present study suggest, for the first time across a large sample, that pupil dilation patterns are significant indicators of sexual orientation. As opposed to previous measures of sexuality, the possible benefits of pupil dilation include a similarity of assessment across the sexes, its potential to reach a wide range of participants, and its ability to capture automatic responses. The assessment of pupil dilation can therefore complement other physiological and self-report measures of sexual orientation.
